# Perceptions Pertaining to Clinical Depression in Karachi, Pakistan

**DOI:** 10.7759/cureus.5094

**Published:** 2019-07-07

**Authors:** Maheen Nisar, Rubaab M Mohammad, Sani Fatima, Preet R Shaikh, Mehroze Rehman

**Affiliations:** 1 Biochemistry, Ziauddin University Hospital, Karachi, PAK; 2 Community Health Sciences, Ziauddin University Hospital, Karachi, PAK

**Keywords:** mental illness, psychiatry, awareness, depression

## Abstract

Introduction

There is a high prevalence of depression in developing countries, and low mental health literacy has been hypothesized as one of the main causes of increasing rates of mental illness in a population. This study aimed to capture an image of the current attitude and perceptions towards victims of clinical depression in Pakistan and to assess the impact of those beliefs.

Methods

A cross-sectional study was conducted with 400 people, chosen through non-probability consecutive sampling, from Karachi, Pakistan. A questionnaire was designed to evaluate the overall perception of depression including its causes, manifestations and treatment options. Descriptive statistics were used and p-values less than 0.05 calculated using the chi-square test were considered significant.

Results

Most of the participants comprehended depression as a natural feeling of sadness rather than a mental disorder. The vast majority cited increased stress (72.2%) and physical/ emotional trauma (51.3%) as the main causes of depression. The most popularly associated symptoms were sadness (53.3%), irritability (53.3%), inability to perform daily tasks (52.8%), and changes in sleeping patterns (52%). Participants believed depression to be best treated by talking to someone trustworthy (59.5%), praying to God (56.5%) and consulting a psychologist/psychiatrist (52.3%). There was a significant association between the participants’ level of education and their perception of clinical depression (p=0.026).

Conclusion

Our study showed a skewed perception of depression with the majority only acknowledging it as a natural feeling of sadness. However, stress was seen as a major perpetrator and the importance of a good support system was acknowledged by most participants. Level of education was revealed to be the most important factor that influenced these beliefs. Effective community-based programs and policies based on these public views will help develop an accessible and autonomous support system for patients with mental illnesses.

## Introduction

More than 20 million Pakistanis (10% of the country’s population) suffer from some form of mental health condition. The full gravity of this situation comes to light with the realization that Pakistan has one of the lowest psychiatrist-to-person ratios in the world. According to the WHO, only 400 psychiatrists and five psychiatric hospitals exist within the entire country for a population exceeding 180 million people [1].

It has been discerned that low mental health literacy could be one of the main causes of high rates of mental illness in a population. Mental health literacy has been defined by Jorm et al. as “knowledge and beliefs about mental disorders which aid their recognition, management or prevention” [2]. People suffering from mental illness also have to bear the prejudices associated with their condition and this stigma is prevalent amongst the Pakistani people [3]. Knowledge of the public’s perception of mental illness is vital towards establishing successful programs to eliminate them [4]. This must be done along with an evaluation of the norms, beliefs, and customs within the respective cultural environment. In Pakistan beliefs in black magic, the evil eye and possession by demons are prevalent. Spiritual leaders have a strong following and are often consulted for solutions to both physical and mental problems [5].

In Pakistan where poverty, unemployment, displacement, and homelessness are continuously on the rise, mental health is also becoming a major public health problem [6]. There are however limited studies based on the perception of the public regarding mental health issues. This paper is therefore designed to give a comprehensive review of mental health literacy amongst the Pakistani public, with regards to clinical depression, with particular emphasis on the causes of the disease, its manifestations in a patient, effective measures of treatment, and help-seeking behaviors.

## Materials and methods

An anonymous self-administered questionnaire was circulated, through non-probability consecutive sampling, to the Clifton, Gulshan, and Saddar areas in Karachi, from August 2018 to October 2018. These areas were chosen because of their varying social and economic infrastructure. The sample size was calculated to be 385 at a 95% confidence interval. The questionnaire was designed in English and also translated to Urdu, the national language of Pakistan. Males and females of all ages, who could comfortably read Urdu or English, were included in this study. The study was approved by the Ethics Review Committee of Ziauddin Medical University and informed written consent was obtained from all respondents before their involvement in the survey.

The first section of the questionnaire addressed the demographic information of the participants. The second section examined the participant's overall perception of depression as a mental condition along with its prevalence in the country. Section three asked further exploratory questions about depression including the signs and symptoms of the disease, the reasons for being depressed, the best methods of treatment, and the main reasons why a depressed person may avoid seeking help. Section four asked about personal experience with clinical depression. The data were entered and analyzed using IBM Statistical Package for the Social Sciences 20.0 (SPSS 20, IBM, Armonk, NY, USA) and frequencies were calculated using descriptive statistics. The chi-squared test was used to find associations of demographic variables with knowledge/attitudes towards depression and personal experience with depression with p values < 0.05 taken as significant. 

## Results

A total of 400 people were enrolled in the study out of which 61.5% were female and 38.5% were male. The mean age of participants was 28.53 ± 13.282 years. Most of them belonged to the Muhajir (26.6%), Punjabi (21.3%), and Sindhi (20.8%) communities. Postgraduates and graduates made up 20.5% and 32% of our subjects respectively. At the time of the survey, 39.4% were studying at intermediate/A level and 5.3% were at matriculation/O level. Most of the responses (50.1%) were from participants with a monthly income of less than 25,000 PKR, followed by those with a monthly income greater than 100,000 PKR (24.1%).

While 45.8% of the participants accurately perceived the prevalence of depression to be high, affecting one out of every four people, 45.5% of them believed it to be moderate, affecting one out of 50 people and 8.3% perceived it to be low, affecting one out of every hundred people. When asked about how they defined depression, 40% of the participants comprehended it as a natural feeling of sadness and 37.8% as a mental disorder, while 12.3% believed it to be an imaginary artifact.

**Figure 1:**
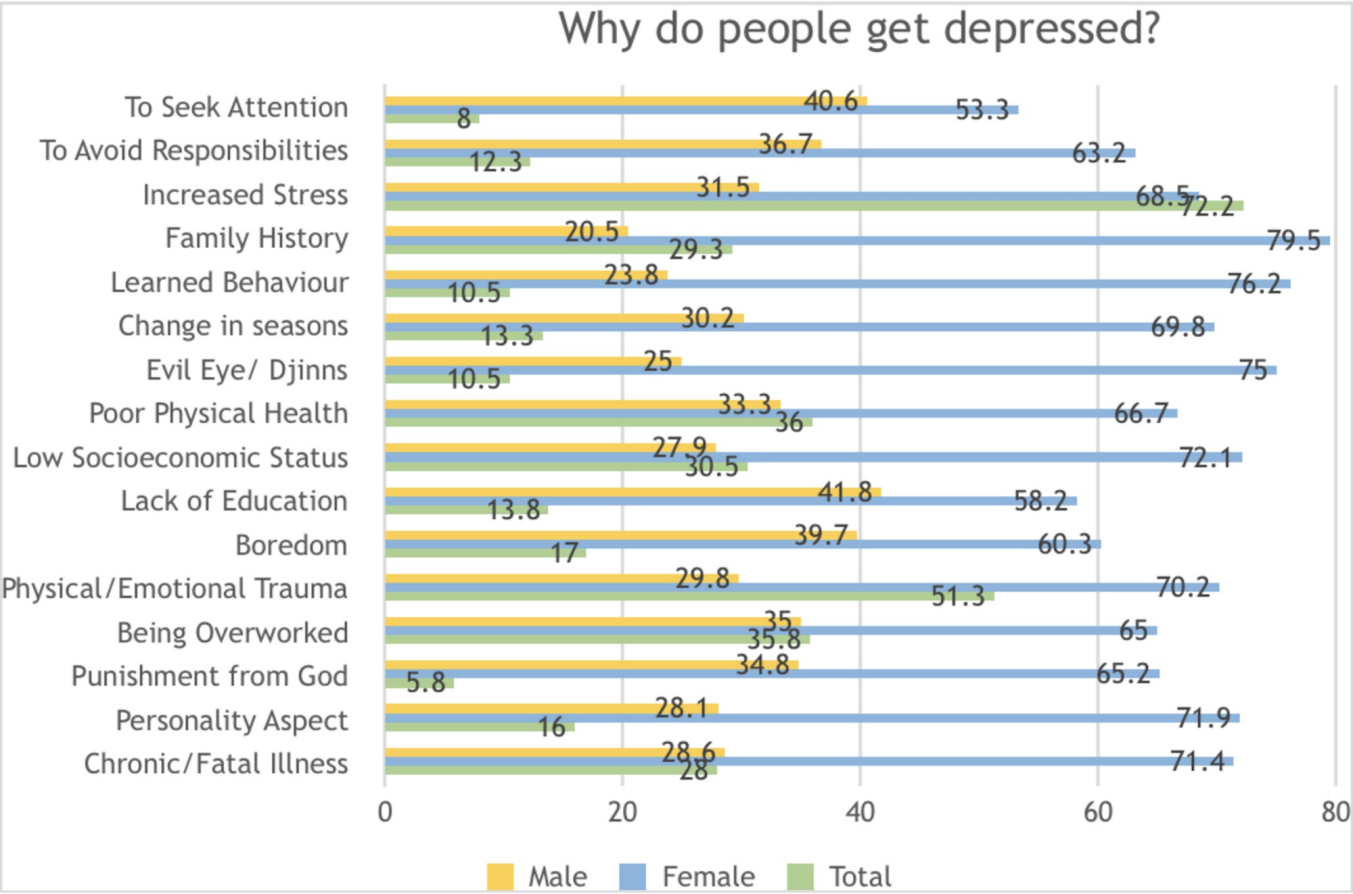
Knowledge and perceptions regarding the causes of clinical depression amongst the general population in Karachi, Pakistan 2018-2019

In terms of causes of depression, increased stress (72.2%), physical/emotional trauma (51.3%), poor physical health (36%), being overworked (36.8%), low socioeconomic status (30.5%), and family history (29.3%) were amongst the most frequently cited reasons. Only 13.3% identified a change of seasons to influence depression. Around 10.5% believed djinns and the evil eye to have an effect and 5.8% reported depression to be a result of God’s punishment. Differing patterns were seen amongst men and women, with men more inclined to attribute a lack of education and a tendency to seek attention as reasons for a person’s depression, while women identified previous family history, depression as learned behavior and evil eye/djinns as the most popular reasons (Figure 1).

**Figure 2:**
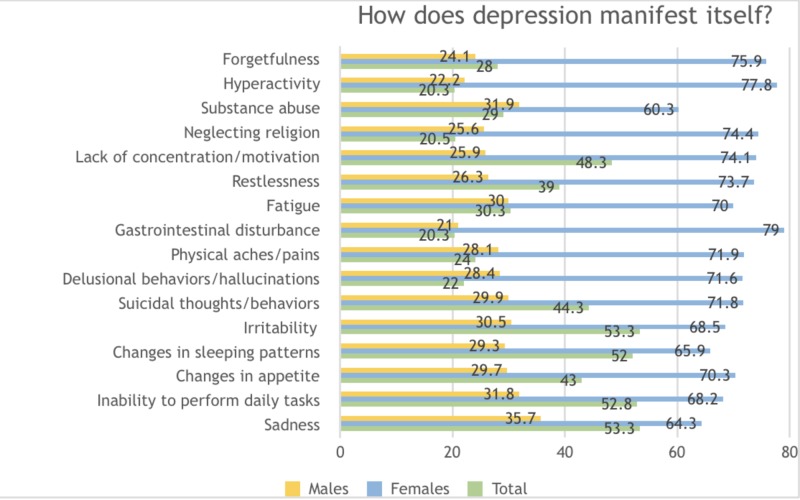
Knowledge and perceptions regarding the manifestation of depression amongst the general population in Karachi, Pakistan 2018-2019

Symptoms were mostly described as sadness (53.3%), irritability (53.3%), inability to perform daily tasks (52.8%), changes in sleeping patterns (52%), lack of concentration/motivation (48.3%), suicidal thoughts/behaviors (44.3%), and changes in appetite (43%) amongst others. Hyperactivity (20.3%), physical aches/pains (24%) and neglect of religion (20.5%) were the least likely to be identified by participants as manifestations of depression. Men were more likely to identify sadness, substance abuse, irritability, fatigue and inability to perform daily tasks as manifestations while women mostly appreciated gastrointestinal disturbances, hyperactivity, and forgetfulness as indications of depression (Figure 2).

**Figure 3:**
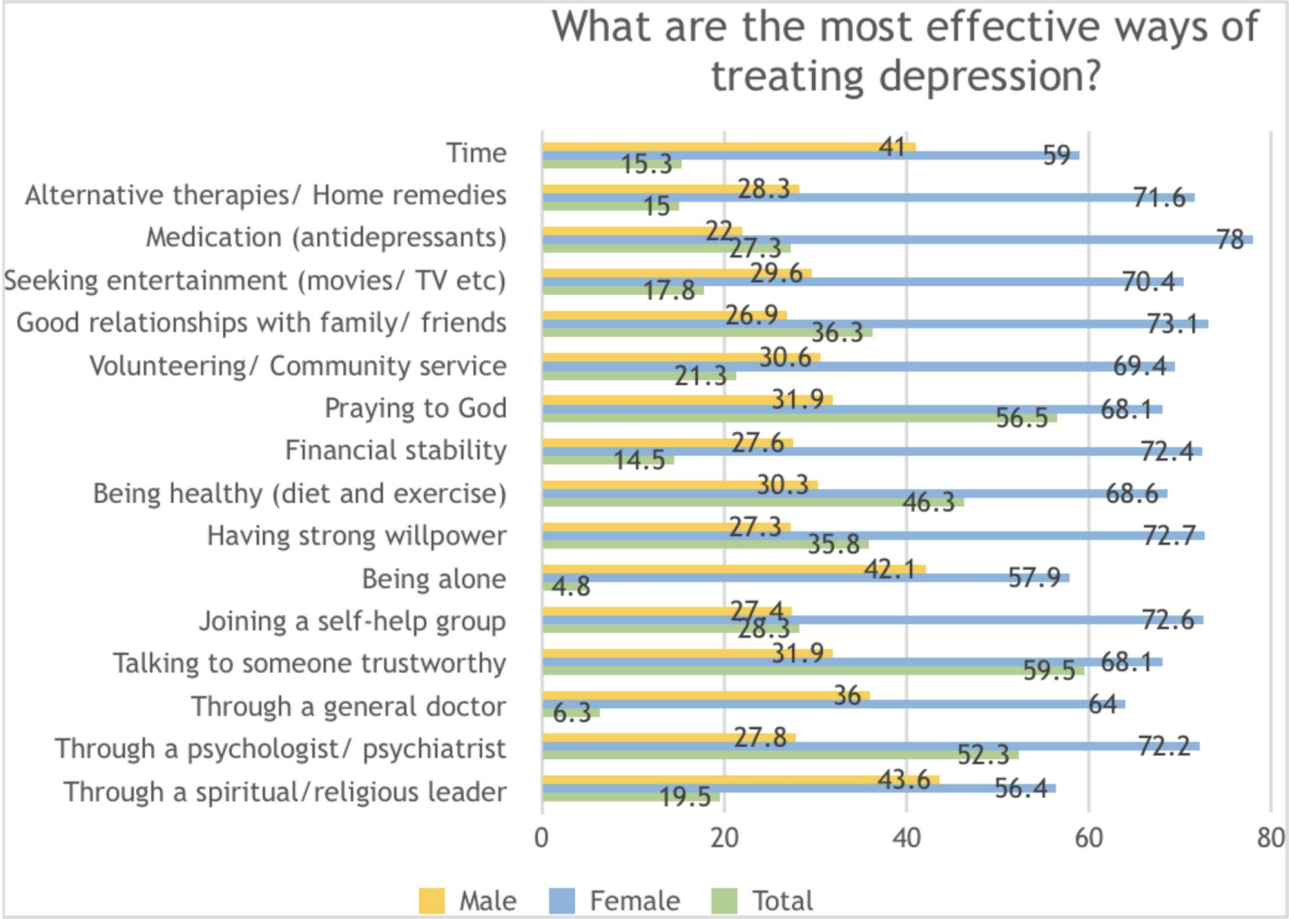
Knowledge and perceptions regarding effective treatments of clinical depression amongst the general population in Karachi, Pakistan 2018-2019

Participants believed depression to be best treated by talking to someone trustworthy (59.5%), praying to God (56.5%), consulting a psychologist/psychiatrist (52.3%), being healthy (46.3%), having good relationships with family/friends (36.3%), and having a strong willpower (35.8%) amongst other factors. While females overwhelmingly proposed medication (such as with antidepressants) as an effective method of treatment, the most popular answers amongst men were seeking help from religious/ spiritual leaders, being alone during one’s depressive phase and to let time do the healing (Figure 3).

**Figure 4:**
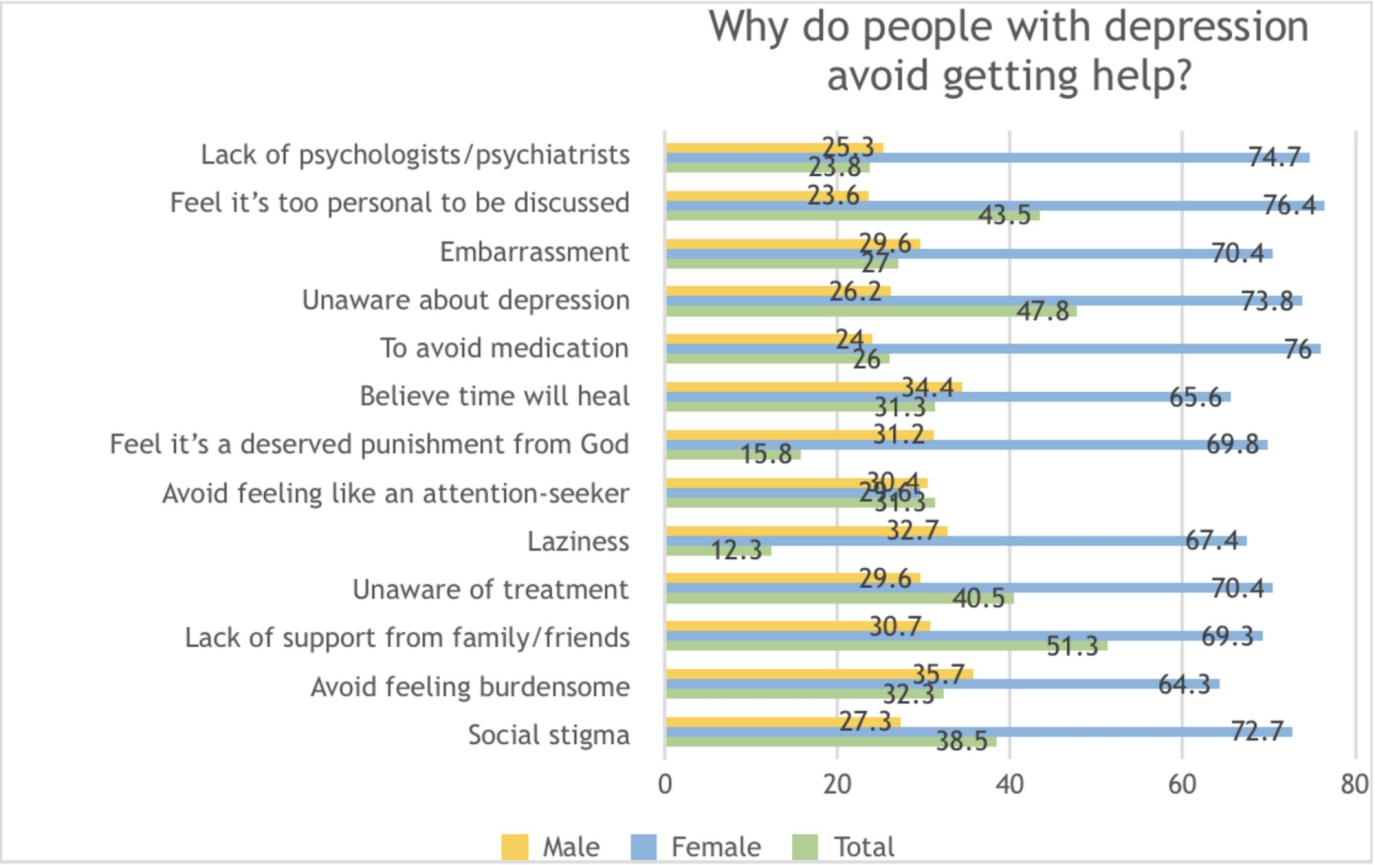
Knowledge and perceptions of why depressive patients avoid seeking help, amongst the general population in Karachi, Pakistan 2018-2019

Participants believed some of the reasons why depressed people avoided getting help were lack of support from family/friends (51.3%), inability to identify their condition as depression (47.8%), feeling it is too personal a matter to be discussed (43.5%), being unaware of the availability of treatment (40.5%), and social stigma (38.5%) (Figure 4).

There was a significant association between a subject’s education and how they perceived depression (p=0.026). Subjects who had been educated at the Intermediate/A level and graduate-level were more likely to perceive depression as a mental disorder, whereas postgraduates were more likely to see it as a natural feeling of sadness. Gender, ethnicity, and income did not influence how depression was perceived (Table 1).

**Table 1:** Association between the perception of depression and demographic profile of participants in Karachi 2017-2018. *P value is significant

Characteristics	What is depression?				
	A mental disorder	A natural feeling of sadness	A figment of the imagination	Total No.	P value
Gender					
Male	48	64	22	134	0.152
Female	103	95	27	225	
Ethnicity					
Sindhi	28	30	16	74	0.319
Muhajir	46	43	12	101	
Pashtun	9	7	2	18	
Baloch	6	4	4	14	
Punjabi	36	39	7	82	
Other	27	37	8	72	
Education					
Primary School	2	5	1	8	0.026*
Matric/O-Level	2	16	2	20	
Inter/A-Level	67	57	22	146	
Graduate	58	49	13	120	
Post Graduate	23	33	11	67	
Monthly Income					
<25,000	61	46	13	120	0.296
25,000-50,000	11	14	5	30	
50,000-75,000	6	3	1	10	
75,000-1 lac	13	6	2	21	
>1 lac	35	22	1	58	

Of the participants, 75.9% had some form of personal experience with clinical depression and 61.5% of those had sought help. Around 62% of the participants had received some form of mental health education and 95.4% thought mental health awareness should be added to the curriculum in schools. A significant association (p=0.006) was found between gender and the subject’s education on depression (Table 2).

**Table 2:** Association of gender with practices and perceptions about depression, in Karachi 2017-2018 *P value is significant

Practices (yes)	Total N (%)	Male N (%)	Female N (%)	p-value
Have you or someone you know ever been clinically depressed?	298 (75.9)	112 (28)	186 (46.5)	0.621
If yes, did they seek help?	201 (61.5)	78 (19.5)	123 (30.8)	0.638
Have you ever received some form of education on mental health? (books, media, etc.)	241 (62)	79 (19.8)	162 (40.5)	0.006*
Do you think mental health awareness should be added to the school curriculum?	374 (95.4)	142 (35.5)	232 (58)	0.79

## Discussion

Depression is a debilitating mood disorder with a worldwide prevalence estimated at 4.4% [7]. Prevalence estimates in Pakistan range from 22% to 60%, with estimates in Karachi (a populous city of 14.9 million) averaging at 47% [8]. The high rates of depression can be attributed to a lack of ‘mental health literacy’ as hypothesized by Jorm et al [2]. In Pakistan, the biomedical aspect of mental disorders is largely ignored, and they are often simplified as a natural consequence of stress-inducing scenarios. Hence Western models of treatment, which are largely based on an understanding of depression as a disease requiring medical or psychological intervention, are difficult to implement in developing countries. The cultural differences make treatments such as psychotherapy, which involve detailed discussions with therapists, limited in success [9]. To improve current treatment regimens it is necessary to have an understanding of the way mental disorders are perceived within a population and with this approach in mind, we reviewed the current public awareness and education on depressive disease within Karachi.

Low socioeconomic status (SES), poor physical health, and the associated psychosocial burdens were amongst the factors most commonly chosen by participants as causes of depression. These results tie in with previous literature. Multiple studies conducted worldwide have found a significant association between poverty and mental health disorders [5]. In a study done on self-harm patients at a tertiary care hospital in Karachi, financial stressors accounted for 70% of the cases. The ‘inverse-care-law’ phenomenon suggests that those of a lower SES receive less health care as compared to those of a higher SES [10]. Pakistan has only 400 psychiatrists and five psychiatric hospitals, with most of them located in urban areas. Considering that the rural areas comprise of 60.5% of the nation’s population of 202 million, access to mental healthcare in rural areas becomes increasingly challenging [2]. 

Good quality healthcare in Pakistan is mainly provided by the private sector and requires patients to pay out-of-pocket. One study approximated the economic burden on an individual wishing to receive treatment from private psychiatric clinics to be above 3,133 PKR per month ($22.58) when 65% of the population earn 5,000 PKR ($36.04) per month [11]. This economic disparity is also highlighted in our study with the majority of participants reporting a monthly income of less than 25,000 PKR per month ($180). Mental health treatment is then seen as unsustainable, making it easier to avoid or ignore symptoms.

Our survey showed the differences in participants’ understanding of depression to be correlated with their monthly income. Results showed that from those of the lowest monthly income 50.8% of participants defined depression as a mental disorder and 10.8% as something imaginary. From those of the highest monthly income, the respective percentages for the same question were 60.3% and 1.7%. A lower SES is often directly proportional to a lack of education, which has also in turn been correlated with depression [6, 12]. In Pakistan cities with the highest suicide rates also have a prevalent low literacy rate, such as Rawalpindi with 2.86 suicides every 100,000 people per year and a literacy rate of 61% in the corresponding province [13-14].

Participants educated at the intermediate/A Level and graduate-level were more likely to perceive depression as a mental disorder. A study on health-seeking behaviors noted that graduates were more self-aware and in turn more likely to reach out for help as opposed to non-graduates [15]. Education is known to improve one’s coping mechanisms, as it promotes increased autonomy and raises self-esteem. Keeping this study in mind, our results were unexpected as most post-graduates perceived depression as a natural feeling of sadness instead of a mental disorder. This could be ascertained to the corresponding age-group of post-graduates, as older adults in society seem to have more traditional and inflexible mindsets over controversial topics. This was also seen in a study conducted on Korean Americans where the older age group saw depression as a sign of personal weakness, whereas younger age groups accepted depression as a medical condition requiring treatment [14].

Physical and/or emotional traumas were also perceived as a major cause of depression, especially by women. Domestic violence in Pakistan is an endemic social and public health problem. A previous study reports that 72% of physically abused women admit to being anxious/depressed [8]. Another common morbidity amongst females is symptoms of adjustment issues, possibly owing to arranged marriages and turbulent relations with in-laws [12]. 

The universal symptoms of depression are reported to be a lack of energy, insomnia, difficulty in concentrating, and suicidal ideation [16]. The majority of the participants in our study had a similar perception of the manifestations of depression. Cultural differences in the presentation have been studied, with non-western countries reporting more somatic symptoms such as pain, sleep disturbance, and fatigue. An explanation for the high rate of somatization points towards societal disapproval of expressing negative emotions. Islamic countries also frequently reported feelings of guilt associated with depression, often owing to religious influences [16]. In our results, neglect of religion was identified by 20.5% of the participants as a manifestation of depression. A majority of participants also expressed religious sentiments when asked about the appropriate treatment of depression with 56.5% choosing ‘praying to God’ as an option and 19.5% choosing contact with a ‘spiritual/religious leader’ as an effective means of treatment.

The lack of awareness of the biopsychosocial causes of mental illness in developing countries often leads to an increased belief in poor mental health being related to supernatural causes, black magic, evil eye as well as a punishment from God [17]. Some of our participants expressed similar views, attributing depression to be the work of djinns/evil eye or a manifestation of God’s punishment, albeit these views accounted for only 10.5% and 5.8% respectively. This perception is consistent with previous studies on acceptable treatment options. In a rural community, supernatural, religious and local approaches, such as special diets and tonics, are heavily relied upon. In certain cases, seeing a local doctor was acceptable but psychiatrists and medications were not with the general belief being that mental illness was best treated in isolation [2, 16]. A lack of financial resources also increases the popularity of seeking alternative treatments for mental health [2]. 

Our results reflected that talking to someone trustworthy and having good relationships with family and friends were seen as effective treatment options. Interestingly, a lack of support and social stigma were also commonly chosen as reasons for why individuals avoid seeking help. This is a dilemma too often faced by those suffering from mental illnesses in Pakistan, as speaking on mental health is considered a social taboo [18]. Those suffering from mental illnesses are publicly mocked in Pakistan, and patients are often labeled as “pagal” (crazy). Cases of discrimination against mental health patients are often reported, with many patients describing being isolated at social gatherings [18]. 

Discrimination against mental health patients has also been reported in medical settings [19]. It is seen that physicians are often reluctant to diagnose someone as depressed [12]. Medical colleges are described as ‘breeding grounds for discrimination’ with many not offering comprehensive courses and clinical rotations in psychiatry [20-21]. As a result, Pakistan is estimated to have one psychiatrist for every 10,000 people, who, as previously mentioned are largely located in urban areas [1]. Our results reflected this gap in accessibility of treatment as ‘inability to diagnose depression’ and ‘unaware of treatment’ were listed by participants as major reasons why depressed people would not seek help.

A greater number of women reported personal experiences with depression, as well as education on the matter, as opposed to men. The rate of depression is higher amongst women, with factors such as a lack of mobility, high infant and maternal mortality rates, tense family relations and boredom having been listed as possible causative factors [1, 22]. The societal pressure against expressing negative emotions in men in eastern culture could account for these results. A difference in approaches to treatment has also been noted with women having a lower return rate, especially if the period between sessions exceeded more than one day [22]. This can be attributed to the fact that Pakistani men have more gender-mobility, whereas women often need to seek permission from their family before leaving the house and may be required to be accompanied. 

The treatment gap for mental health in developing countries is estimated at 90% by WHO [23]. In Pakistan, this huge gap can be attributed to social stigma, lack of education, and limited financial resources amongst other factors. Due to a lack of health insurance policies and programs, as well as the volatile political climate, NGOs play an essential role in increasing awareness and accessibility of treatment to mental illnesses [9]. Their roles rely on following a community-oriented approach catering to the perceptions and beliefs of a specific populace. Studies on the general views of the population are therefore necessary for such individualized community programs to be effective. The majority of our participants believed mental health awareness should be added to school curriculums. Further research on the effective implementation of such programs is needed, along with how they can be transferred to professional environments and the general public. Information such as this will aid NGOs and clinics in their approach to mental health care and work towards creating a supportive environment for patients, increasing their sense of autonomy. Moreover, policies regarding mental health need to be regulated to improve the remarkably low patient-to-psychiatrist ratio in the country.

## Conclusions

Our study showed a skewed perception of depression, with the majority only acknowledging it as a natural feeling of sadness. However, stress was seen as a major perpetrator and the importance of a good support system was acknowledged by most participants. The level of education was revealed to be the most important factor that influenced these beliefs. Effective community-based programs and policies based on these views held by the public will help develop an accessible and autonomous support system for patients with mental illnesses. 
